# Identification of PROS1 as a Novel Candidate Gene for Juvenile Retinitis Pigmentosa

**DOI:** 10.22088/IJMCM.BUMS.8.3.179

**Published:** 2019

**Authors:** Ata Bushehri, Davood Zare-Abdollahi, Afagh Alavi, Alireza Dehghani, Mohammadreza Mousavimikala, Hamid Reza Khorram Khorshid

**Affiliations:** 1 *Genetics Research Center, University of Social Welfare and Rehabilitation Sciences, Tehran, Iran. *; 2 *Department of Ophthalmology, Eye Research Center, Isfahan University of Medical Sciences, Isfahan, Iran.*; 3 *Department of Ophthalmology, Tabriz University of Medical Sciences, Tabriz, Iran. *

**Keywords:** Retinitis pigmentosa, RP, PROS1, protein S, TAM receptor, efferocytosis, apoptosis

## Abstract

Homozygous mutations of PROS1, encoding vitamin K-dependent protein S (PS), have been reported so far to be associated with purpura fulminans, a characteristic fatal venous thromboembolic disorder. The current work for the first time reports the clinical phenotype in patients with juvenile retinitis pigmentosa harboring a novel likely pathogenic variant in thePROS1 gene. Whole-exome sequencing was performed on probands of a cohort with inherited retinal disease. Detailed phenotyping was performed, including clinical evaluation, electroretinography, fundus photography and spectral-domain optical coherence tomography. Analysis of whole-exome and Sanger sequencing led to the identification of a homozygous missense substitution (c.G122C:p.R41P) in PROS1 in affected individuals from two unrelated consanguineous families of Persian origin which had classic retinitis pigmentosa with no history of venous thromboembolic disorder. This variant was segregated, fully congruous with the phenotype in all family members. Consistently, none of 1000 unrelated healthy individuals from the same population carried the mentioned variant, according to Iranian national genome database (Iranome) and additional in-house exome control data. This study provides inaugural clinical traces for different role of PS as a ligand for TAM receptor-mediated efferocytosis at the retinal pigmented epithelium; the R41P variant may affect proper folding of PS needed for γ-carboxylation and extra-cellular secretion. That conformational change may also lead to defective apoptotic cell phagocytosis resulting in postnatal degeneration of photoreceptors.

Retinitis Pigmentosa (RP) belongs to a tremendously heterogeneous group of inher-ited retinal degenerations/ dystrophies (IRD), affec-ting rod and cone photoreceptors. Delayed dark adaptation as an early symptom, night blindness, progressive photophobia, gradual deterioration of peripheral vision, and finally inexorable macular involvement, is the typical course of the disease (1). In accordance with its widespread presence of 1 in 4000, there are more than 1.5 million affected individuals worldwide.. RPs could be genetically transmitted in all modes of inheritance,whereby autosomal dominant (adRP) accounts for 30-40% while autosomal recessive (arRP) and X-linked (xlRP) patterns include 50-60% and 5-15% of the cases, respectively(2). Upon fundus examination, RP usually appears with narrowed retinal arterioles, optic disc pallor and peripheral intraretinal pigment mottling, despite extensive clinical variability in terms of initial symptoms, age of onset, visual field constriction pattern (rod vs cone involvement), presence of macular lesions, and systemic manifest-ations. In clinical practice, advancement of RP is measured by an electroretinogram (ERG) in which the electrical responses of photoreceptor cells are gradually reduced and eventually non-recordable (3). Optical coherence tomography (OCT) and fundus autofluorescence (FAF) imaging, at later stages, show an intensifying loss of outer retinal layers and altered lipofuscin scattering in a characteristic pattern resulting in retinal pigmented epithelium (RPE)/ Bruch’s membrane complex thinning(4).The RPE has a task in nourishing retinal light sensitive cells, phagocytosis of outer segment (OS) membrane debris and clearance of apoptotic cells, a phenomenon known as efferocytosis. Photoreceptors’ oxygen consumption during photo-transduction cascade (4, 5), produces discrete deposition of lipofuscin and other remnants made of damaged proteins and demolished cholesterols by free radicals along the cuticular layer of Bruch’s membrane where residues of massive expenditure of OS disks reside (4). Metabolic burden due to aggregation of oxidized and potentially toxic materials plus accumulation of immunogenic shed photoreceptor OS on the underlying RPE cells is capable of inducing apoptosis in neighboring cells, leading consequently to inflammation spread(4-6). The hallmark of such chaotic microenvironment is the emersion of the cytoplasmic pro- apoptotic proteins which activates specific apoptotic proteases (7). In fact, gradual rod- cone dysfunction is an inevitable outcome of apoptosis induction of RPE cells unless a proper protective system against such inducers is in place (8).

The protein S (PS) gene (PROS1) allocates the composition of vitamin K dependent anticoagulant plasma PS (9, 10), which functions as a cofactor of activated protein C in the degradation of coagulation factors Va and VIIIa, as well as factor Xa inhibitor via direct binding to the factors Xa and Va. Given these coagulation regulatory functions, it is not surprising that PROS1 variants, with loss of function effect, might be associated with an increased risk of thrombosis. So that, heterozygotes are at risk of recurrent venous thrombosis and cardiovascular accidents during adolescence, while homozygotes suffer from purpura fulminans during infancy necessitating fresh frozen plasma administration (11). However, in line with the previous commentaries, PROS1 encodes a peculiar and biologically relevant ligand of Tyro3/Axl/Mer (TAM) receptors which yields pleiotropic executions (12), such as promoting efferocytosis meticulously in high oxygen consumptive tissues like RPE cells. It is hypothesized that the proper visual function requires the correct functioning of TAM receptors and their downstream pathways where PS plays a role as well.

In this study, a novel homozygous missense variant in PROS1 gene in two unrelated consanguineous families associated with non-syndromic RP was identified. The supposedly deleterious variant p.R41P, consistent with an autosomal recessive mode of inheritance, co-segregated with the phenotype among all members of both families. We propose that there is a possible link between a patient suffering from IRD and specific constitutional PROS1 mutations.

## Patients and methods

**Patients, phenotypic and clinical data**

**Family A:** The proband was a 62 year old male, descent of first cousin healthy parents with 5 physically fit and intact siblings, originating from a remote village from the province of Yazd, central Iran. He was ascertained after her daughter was referred to seek pre-marriage genetic counseling. Suffering from nyctalopia for almost 50 years, he, at his initial visit, was just able to distinguish light from dark. The early symptom was exhibiting impaired dark adaptation with the onset at the age of 9 (Table 1).

**Fig. 1:**
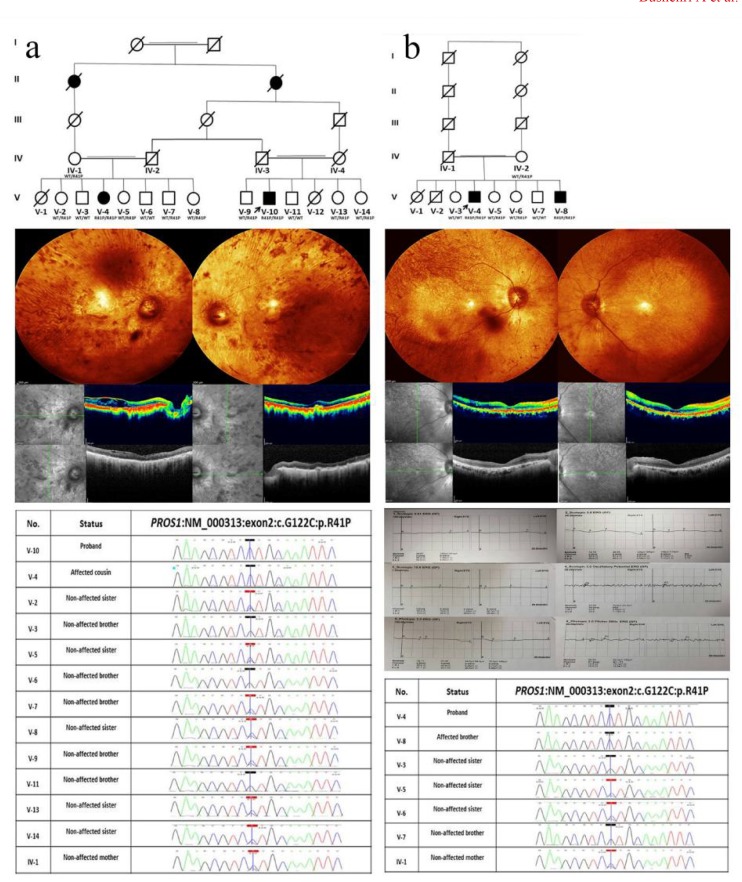
**Pedigree of families, retinal imaging and sequencing electropherograms of PROS1-related RP. a:** pedigree chart of family A with R41P PROS1 mutation. Retinal imaging of patient V-10 (proband) from family A. Central fundus photographs of both eyes: visible choroid arteries due to extreme RPE loss, grievous arteriolar narrowing, waxy pallor optic disc, bone-spicule pigmentary changes, reduced foveal reflex, also choroidal folds and visible sclera in nasal quadrant of the left eye are more prominent. Spectral domain optical coherence tomography (SD-OCT) of the macula; right eye: Epi-retinal membrane, retinal fold and cystoids edema of the macula, left eye: normal retinal structure. Results of family A members’ Sanger sequencing electropherograms identifying the missense mutation, c. G122C, are compatible with their phenotypes. **b:** pedigree chart of family B. Retinal imaging of patient V-4 (proband) from family B; fundus photographs of both eyes: RPE loss, arteriolar narrowing, waxy pallor optic disc, bone-spicule pigmentary changes, and reduced foveal reflex. SD-OCT of both eyes: atrophy of the inner retinal layers in the peripheral macula. Full-field ERG of proband of family A showed an advanced RP pattern of generalized dysfunction: a flat rod-driven response of on bipolar cells (dark-adapted 0.01 ERG); almost undetectable rod dominated combined responses from both photoreceptors and bipolar cells (dark-adapted 3.0 ERG); non-responsive dark-adapted 10.0 ERG with enhanced a-waves reflecting combined photoreceptor function; significantly reduced amacrine cell responses (scotopic 3.0 oscillatory potential ERG); completely extinguished cone-pathway-driven responses on phototopic 3.0 ERG and markedly reduced sensitive light adapted response (30 Hz flicker ERG). Results of family B members’ Sanger sequencing electropherograms

The history of sequential night blindness,

gradual limitation of peripheral vision, defects in color vision and then development of central scotoma was in place, correspondingly compatible with the diagnosis of RP. There were other affected individuals with identical disorder in the proband’s extended family, including his existing cousin who was recruited in this study as well. The pedigree of the family was clearly suited to recessive mode of inheritance (Fig.1a). In terms of ophthalmologic examination, fundoscopy revealed severely attenuated retinal blood vessels symmetrically, extremely dreadful RPE loss in which choroid arteries and sclera had become visible to some extent. Moreover, fiddling formation of bone corpuscular lesion and reduced foveal reflex were further findings. Also, optical coherence tomogr-aphy (OCT) disclosed retinal fold at both eyes along with epi-retinal membrane at the right eye (Fig.1a). Notwithstanding normal anterior chamber segment upon slit lamp biomicroscopy, there were signs of posterior subcapsular cataract (PSC) in the lens and dust-like particles in the vitreous body. Besides, full-field ERG was non-recordable (Fig.1 b). All findings were in favor of RP.

**Table 1:** Clinical description of the patients

**Family.Subject ID/Gender. Age at last review**	**A.V-10/M.62**	**A.V-4/F.60**	**B.V-4/M.50**	**B.V-8/M.42**
**Symptom (Onset)**	IDA (9) NB (10) TVF (mid 20s) VAD (28) CS (28) Photophobia Photopsia	NB (11) TVF (mid 20s) VAD (late 20s) CS (30) Photophobia Photopsia	NB (early teen ages) TVF (early 20s) VAD (mid 20s) CS (27) Photophobia Photopsia	NB (11) TVF (early 20s) VAD (26) CS (26) Photophobia Photopsia
**VF to confrontation**	NA	NA	5°-10° Central	10°-20° Central
**BCVA**	OD: LP OS: LProj	OD: LProj OS: LProj	OD: 0.001 (HM@2’) OS: 0.001 (HM@2’)	OD: 0.1 OS: 0.1
**Fundoscopy**	VDLP Severe RVA Intensive RL Fiddling BSC WODP AFR	VDLP Severe RVA Intensive RL Fiddling BSC WODP AFR	VDLP RVA Diffuse RL Slight BSC WODP AFR	VDLP RVA RL Slight BSC ODP AFR
**Additional findings**	PSC CV:NA RE: Plano Fixation: NA	PSC CV: NA RE: Plano Fixation: NA	PSC CV: Achromat RE: Plano Fixation: NA	PSC CV: Protanopia RE: Plano Fixation: No
**Pattern of functional disorder**	RCD	RCD	RCD	RCD
**OCT**	Retinal atrophy Epi-retinal membrane (OD)	Not performed	Retinal atrophy	Not performed
**ERG**	Non-recordable	Not performed	Non-recordable	Not performed

AFR: absent foveal reflex; BCVA: best corrected visual acuity; BSC: bone spicule configuration; CS: central scotoma; CV: color vision; HM: hand motion; IDA: impaired dark adaptation; LProj: recognition of light projection; LP: light perception; NA: not applicable; NB: night blindness; OCT: optical coherence tomography; ODP: optic disc pallor; OD: right eye; OS: left eye; PSC: posterior sub-capsular cataract; RE: refractive errors; RL: RPE loss; RVA: retinal vessels attenuation; TVF: Tunnel visual field; VAD: visual acuity disturbance; VDLP: vitreous dust like particles; VF: visual field; WODP: waxy optic disc pallor;

**Family B:** Two brothers, aged 50 and 42, were offspring of a third-cousin healthy couple emanating from the province of Kerman, almost 200 miles away from provenance of family A. The subjects were evidently known cases of IRD, in particular rod-cone dystrophy. The notable similarity in the clinical presentations of the affected sibling was remarkable. The disorder started in two affected brothers at the age of 11 with occurrence of night blindness, followed by photophobia and photopsia, led to the visual field, color vision and visual acuity defects discernible at ages of 20, 24, and 26, respectively. The younger brother still enjoys evanescent visual intuition with the visual acuity (VA) of bilaterally 0.1, color vision of protanopia and presence of searching nystagmus, but no visual fixation. Recessively inherited pattern was the most justified mode of genealogy (Fig.1b).The ophthalmologic findings (Fig.1b) were amazingly similar to those of the family A, demonstrating diffuse chorioretinal atrophy. ERG was also non-recordable.

For both family affected members, there was no history of developmental delay, including motor and language capabilities, no sign of other organ in-volvement, such as auditory, nervous, geni-tourinary, and musculoskeletal systems. Therefore, they are all considered to be non-syndromic RP.

**Molecular genetics investigation**

Written informed consent was obtained from all participants based on procedures approved by the research ethics committee of University of Social Welfare and Rehabilitation (USWR), Tehran, Iran, in agreement with declaration of Helsinki (13). Following obtaining 5 ml blood samples from all family members, genomic DNA was extracted using salting out method. Whole-exome sequencing (WES) was performed on two patients, one from each family, starting with the workflow of library construction from purified DNA and exome enrichment with the application of SureSelect Enrichment (Human All Exon V6; Agilent Technologies Inc.). The second step was sequencing of exonic DNA using platform of Illumina HiSeq 4000 to a sufficient coverage of >100. In terms of primary data processing, row sequence reads were aligned to GRCh38 reference genome (https:// www. ncbi.nlm. nih.gov/ grc/ human/ data/) using conventional alignment tools (ELAND, BWA). Concerning secondary data sorting, variants were called and filtered employing quality control criteria (Samtools) to create genome variant call formatted (VCF) files for each sample. Then emanated variants were annotated using ANNOVAR (http://www.openbioinformatics.org/) relying on Ensemblgene and transcript definitions (14).

**Data processing**

Tertiary analysis of the annotated variants (15) was conducted based on consistency with assumed recessive mode of inheritance (homozygous or compound heterozygous variants), function (nons-ense, frameshift indels, stop gain, stop loss, splicing, and missense variants with a nucleotide conservation score of GERP++> +2 and CADD> 20), minor allele frequency (MAF<0.005 against 1000 genomes, ExAC, EVS, gnomAD, Iranian national genome database: Iranome(http://iranome. ir/), containing exome sequencing variants from 800 healthy individuals from eight major ethnic groups in Iran, and our in-house exome data from 200 ethnicity-matched unaffected persons), and reported IRD associated genes using gene-disease associated databases (16).

After gene assessment and variant classific-ation according to the American college of medical genetics and genomics (ACMGG) guidance for variant classification (17),integration of results with patients’ phenotype was implemented with the aim of providing a clinically relevant interpretation of the findings.

Concerning validity, confirmation of candi-date variants and segregation analysis were performed on 4 subjects, 2 mothers and 14 non-affected siblings. With the application of a pair of specifically designed primers flanking the desired variant and by polymerase chain reaction (PCR), the intended fragment of DNA was captured and amplified to be properly sequenced through Sanger method using standard protocols. Regarding designated variant nomenclature in compliance with GenBank Accession number NM_000313, variant was interpreted as novel as it was not previously reported in the literature and absent from dbSNP, EVS, ExAC and gnomAD.

## Results

We identified a homozygous single nucleotide substitution (NM_000313:c.G122C:p.R41P) in exon 2 of the PROS1 gene. It converts arginine (R), which is an amino acid with hydrophilic and amphipathic moiety and contains a highly polar positively charged guandino group (18), to proline (P), a hydrophobic amino acid with an exceptional conformational rigidity owing to the distinctive cyclic structure of its side chain (19).

**Table 2a:** Conserved 18-amino acid propeptide sequences upstream of human vitamin K-dependent (VKD) proteins

**VKD propeptide**	-18	-17	**-16**	-15	-14	-13	-12	-11	**-10**	-9	-8	-7	**-6**	-5	-4	-3	-2	**-1**
**Protein S**	A	N	**F**	L	S	K	Q	Q	**A**	S	Q	V	**L**	V	R	K	R	**R**
**Protein C**	S	V	**F**	S	S	S	E	R	**A**	H	Q	V	**L**	R	I	R	K	**R**
**Factor X**	S	L	**F**	I	R	R	E	Q	**A**	N	N	I	**L**	A	R	V	T	**R**
**Factor IX**	T	V	**F**	L	D	H	E	N	**A**	N	K	I	**L**	N	R	P	K	**R**
**Factor VII**	R	V	**F**	V	T	Q	E	E	**A**	H	G	V	**L**	H	R	R	R	**R**
**Prothrombin**	H	V	**F**	L	A	P	Q	Q	**A**	R	S	L	**L**	Q	R	V	R	**R**
**PRGP1**	R	V	**F**	L	T	G	E	K	**A**	N	S	I	**L**	K	R	Y	P	**R**
**Matrix Gla Protein**	N	P	**F**	I	N	R	R	N	**A**	N	T	F	**I**	S	P	Q	Q	**R**
**Bone Gla Protein**	K	A	**F**	V	S	K	Q	E	**G**	S	E	V	**V**	K	R	P	R	**R**

Highly conserved amino acids (F -16, A -10, L -6 and R -1) are highlighted in yellow.

The nucleotide transversion interchanges a guanine (G) to a cytosine (C) at the position 93646206 on chromosome 3, confirmed by direct sequencing. Intriguingly, this mutation changes the molecular conformation of PS propeptide which is highly conserved among human vitamin K-dependent (VKD) proteins (Table 2a), exactly where the excision of the first 41 amino acids would make the mature 635- amino acid PS release.

Noticeably, the mentioned variant has not been reported previously in gnomAD, ExAC, ESP6500, 1000 Genome and our national genome database (Iranome). Finally, the variant co-segregated with the non-syndromic RP phenotype in both families (Fig. 1 a and b). No other candidate variants were found in WES analysis of family B while the proband of family A also had a previously reported homozygous variant in aconitase 2 (ACO2). ACO2 is strongly associated with infantile cerebellar-retinal degeneration (MIM # 614559) and hence, this variant was excluded indisputably on account of its severity and systemic involv-ement.

## Discussion

RP is an inherited disorder caused by a progressive decrease in rod and cone photoreceptor function. Despite identification of more than 70 known genes responsible for RP, there are still many unknown RP-associated genes yet to be discovered. Our study is the first report showing that a PROS1 mutation is linked to human non-syndromic RP.

**Fig. 2:**
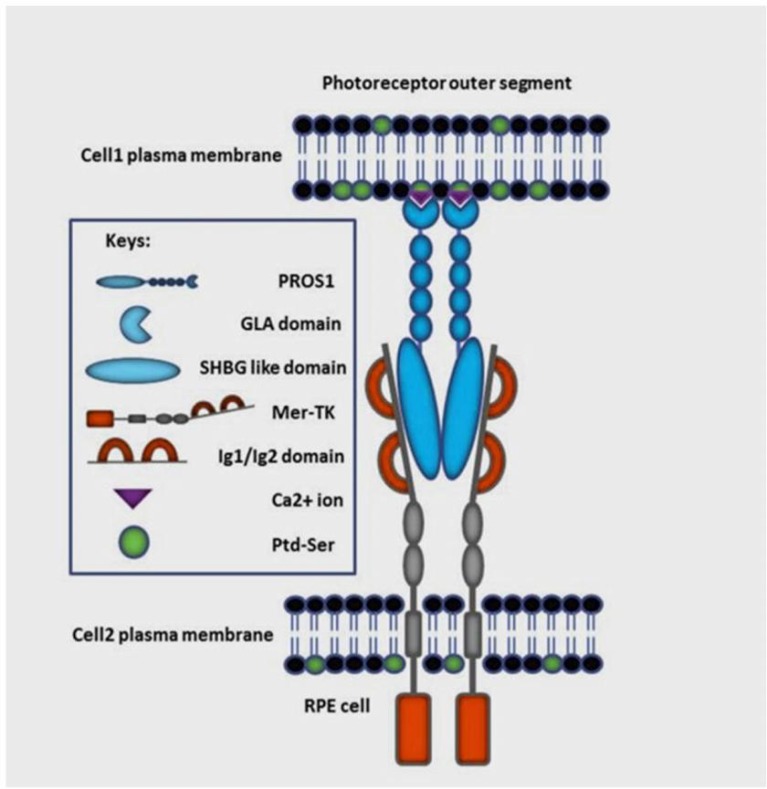
**Immunologically silent phagocytosis (efferocytosis) in the retina via PtdSer-PS-Mer signaling. **PtdSer-PS-MerTK signaling drives phagocytic pruning of just photoreceptor outer segments by RPE cells on regular basis. Binding of PS (blue) to the Mer (grey and red) receptor is mediated by its sex hormone-binding globulin (SHBG)-like domains through the two immunoglobulin-related domains (Ig1/Ig2) of the Mer receptor. The binding of essential Ca^2+^ ions (purple) stabilizes the structure of the GLA domain where it detects the head group of phosphatidylserine (PtdSer) (green). Cell1 represents photoreceptors and cell2 represents RPE phagocytic cells

Basically, the 676 residue single-chain PS is the product of PROS1 gene composed of 15 exons. After synthesis, the PS precursor (preproprotein) goes through two consecutive post-translational modifications to cleave off the first 24 N-terminal amino acids (known as prepeptide or signal peptide) and 25-41 amino acids (propeptide), respectively (20). The critical and highly conserved propeptide (Table 2b) is a foremost attachment spot for carboxylase which works before propeptide cleavage(21). Then, the secretary protein becomes releasable from the cell. The mature PS consists of an N-terminal GLA domain followed by the tenase sensitive region (TSR) which is a thumb loop bridged by a disulfide functional group. Succeeding the TSR, there are four EGF-like domains in tandem tracked by a couple of C-terminal sex hormone-binding globulin (SHBG)-like domain, comprising of two tandem laminin G domains (Fig.2). 

**Table 2b:** High degree of similarity of propeptide of PS among vertebrates

**Propeptide of PS**	-18	-17	-16	-15	-14	-13	-12	-11	-10	-9	-8	-7	-6	-5	-4	-3	-2	-1	Similarity
**Human**	A	N	F	L	S	K	Q	Q	**A**	S	Q	V	**L**	V	**R**	K	**R**	**R**	**100%**
**Chimpanzee**	A	N	F	L	S	K	Q	Q	**A**	S	Q	I	**L**	V	**R**	K	**R**	**R**	**94%**
**Gorilla**	A	N	F	F	S	K	Q	Q	**A**	S	Q	V	**L**	V	**R**	K	**R**	**R**	**94%**
**Marmoset**	A	N	F	L	S	K	Q	Q	**A**	S	Q	V	**L**	I	**R**	K	**R**	**R**	**94%**
**Mouse**	T	N	F	L	S	K	E	R	**A**	S	Q	V	**L**	V	**R**	K	**R**	**R**	**83%**
**Sheep**	A	N	F	L	S	R	Q	H	**A**	S	Q	V	**L**	V	**R**	R	**R**	**R**	**83%**
**Armadillo**	D	S	I	L	S	K	Q	Y	**A**	S	Q	V	**L**	F	**R**	K	**R**	**R**	**72%**
**Chicken**	A	T	F	L	S	H	Q	Y	**A**	S	E	F	**L**	A	**R**	K	**R**	**R**	**67%**
**Chinese turtle**	M	F	L	L	S	Q	Q	Y	**A**	S	E	F	**L**	V	**R**	K	**R**	**R**	**61%**
**Xenopus**	R	T	F	L	S	P	Q	Y	**A**	S	E	F	**L**	N	**R**	R	**R**	**R**	**56%**
**Zebrafish**	Q	R	F	L	P	Q	S	K	**A**	S	E	F	**L**	L	**R**	H	**R**	**R**	**44%**
**Asian bonytongue**	Q	H	F	L	Q	Q	S	T	**A**	L	Q	F	**L**	A	**R**	R	**R**	**R**	**44%**
**Big-finned mudskipper**	S	L	F	L	G	R	S	S	**A**	S	Q	F	**L**	S	**R**	Q	**R**	**R**	**44%**

Highly evolutionary conserved amino acids (A -10, L -6, R -4, -2 and -1) are highlighted in yellow.

Consistent with other vitamin K-dependent proteins, PS has a glutamate-rich motif at the GLA domain to be carboxylated, to develop into its biological active form. In a more precise explanation, γ-carboxylation makes the GLA domain able to bind to Ca^2+^ ions which is necessary for conformational changes needed to interact with negatively charged phospholipids (22, 23). This interaction conducts a unique cellular signaling and macrophage chemotaxis, which is regulated by MerTK-PS pair acting as a phospholipid detector (Fig.2). In other words, activation of MerTK, as one of the most important downstream effectors of the TAM receptors, demands cohesion of the γ-carboxylated N-terminal of the PS to the cellular membrane phospholipid phosphatidylserine (PtdSer) as a signal of irreversible cellular damage (24). Despite its apparent serenity upon quotidian residing at the cytoplasmic face of the plasma membrane, PtdSer is considered a salient signal mediating cellular contact when exposed externally (24, 25). This exoplasmic translocation provides a pruned chemotactic stimulus for macrophages to engulf and vanish metabolically hurt cells suffering from oxidative trauma (24). It is done through exclusive binding of PS SHBG-like domain to the two immunoglobulin-related domains (Ig1/Ig2) of the TAM receptors including MerTK. Dimerization of the receptor as a consequence of the latter binding leads to MerTK phosphorylation. It, in turn, inhibits a chronic inflammatory immune response. Comprehensively, triggering interferon-α receptor / signal transducer and activators 1(IFNAR/STAT1) by PS-TAMR switches on the expression of suppressor of cytokine signaling 1 and 3 (SOCS1/3) which, seriatim, prevents toll-like receptors (TLRs) and other pro-inflammatory pathways (5). That is how PtdSer-PS-MerTK signaling clears apoptotic cells and debris, and prohibits the chronic immune response. Specifically, RPE cells cut off only the edge of photoreceptor OS on every day of life in which the task is dedicated to MerTK signaling pathway. Mutations of MERTKgene, encoding MerTK protein, are known as the cause of inherited RP (RP38; OMIM: 613862) (24). Defect in activation of MerTk results in efferocytosis failure of RPE cells. As revealed, TAM triple knockout mice (TKO) developed visual impairment in adulthood. Intriguingly, the phenotype of TKO infant mice are significantly similar to that of wild types (5). AsTAM receptors expression is dramati-cally increased after birth and kept at high levels in adulthood, it indicates its critical role in postnatal maintenance of visual function(5). In fact, elimination of MerTK phosphorylation causes adult onset retinal degeneration through accum- ulation of debris and subsequent innate immunity inducement (26).

**Fig. 3:**
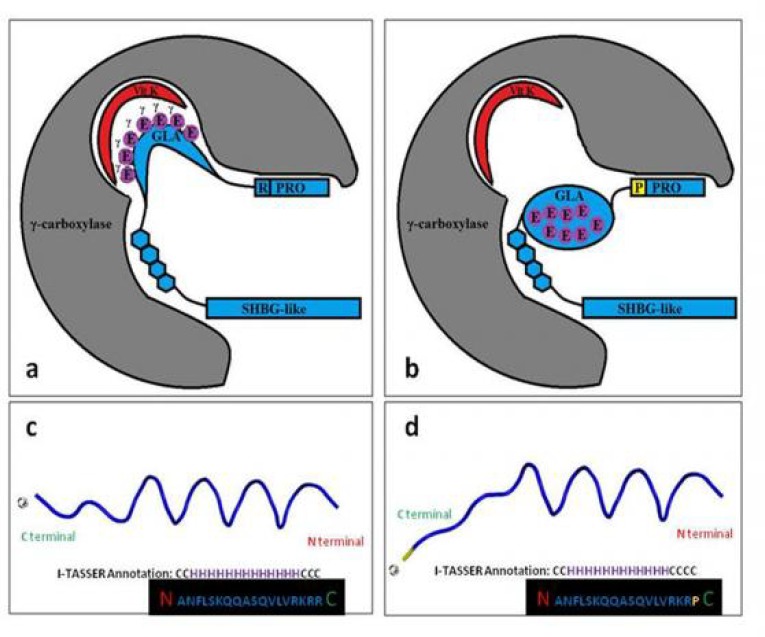
**Effect of p.R41P variant on C-terminal residue of the propeptide sequence.** **a and b:** replacement of the highly conserved R -1 by a P (yellow) at last C-terminal residue of the propeptide (PRO), with respective hydropathicity score of -4.500 and -1.600 (https://web.expasy.org/protscale/pscale/Hphob.Doolittle.html/). This substitution affects proper folding of PS (blue) in ER lumen needed for γ-carboxylation and extra-cellular secretion. Proline as the only amino acid that has a secondary amine attached directly to its side chain disrupts the binding of glutamate residues (purple)  to the reduced vitamin K (red) at the γ-carboxylase (grey) active site by launching a sharp bend. It also interferes in α-helix formation by steric effects and electrostatic repulsion. **c and d:** predicted effect of p.R41P variant on the 3D propeptide of PS structure (using PyMOL molecular visualization system software through manual residue building). Close-up structure model of the amino acid position 41 with blue as wild type and yellow/blue representing the p.R41P mutant. The substitution alters the conformation of the connection loop between two α-helices of the propeptide and the N-terminal of the GLA domain (according to predicted secondary structure from I-TASSER Annotation; https://zhanglab.ccmb.med.umich.edu/I-TASSER/output/S423178/). H: helix; C: coil

In our RP families, the p.R41P variant affects the last C-terminal residue of the propeptide sequence (Fig.3a and b). The propeptide contains γ-carboxylation recognition sequence which not only provides a docking site for the enzyme tied up to the substrate, but also controls allosteric regulation (27). This bridling determines the processivity of the enzyme and subsequently the fulfillment of perfect carboxylation (22).It has been demonstrated that site-directed mutagenesis in the propeptide (lacking -1 to -18 residues) of VKD proteins results in complete failure of carboxylation (28, 29). Furthermore, it is indicated that insertion of highly polarized residues of -1 to -8 could increase affinity of γ-carboxylase to the PS by almost three folds, while inclusion of the hydrophobic part of the propeptide (-8 to -14) elevates significantly the efficacy of carboxylation by 45 times (30). Besides, the C-terminal part of the propeptide also embodies the propeptidase recognition site, crucial for mature PS secretion (31). It has been revealed that arginine substitusion at the position of -1 or -4 prohibits propeptide processing leading to non-functional PKD proteins (31, 32). What is more, an amin (NH2 of arginine) as an active base at the position of -1 is necessary to induce deprotonation to initiate the ionization of reduced vitamin K (22). In fact, this conserved and functionally significant R has been replaced by a P. Altogether; proline due to its unusual conformational rigidity affects the secondary structure of the propeptide needed for γ-carboxylase as well as propeptidase tethering (Fig.3 c and d). En passant, our affected individuals demonstrated no thrombophilic features typical for homozygous PS deficiency which is a severe and fulminant thromboembolic disorder. Concordantly, their coagulometric assays and serum PS levels evaluated within normal range. Hence, we can hypothesize that homozygous p.R41P represents a hypomorphic variant with a mild effect on PS activity as mentioned. 

It has been cleared that the retina and particularly RPE cells have the highest metabolic expenditure among body tissues (33), far higher than brain and kidney in terms of oxygen consumption levels (4, 34). One could speculate that PROS1 leaky mutations result in postnatal blindness in which residual activity of PROS1 suffices for proper function in all organs except the eyes. In other words, inadequate function of PS at demanding RPE, ensuing cumulative failure of MerTK mediated efferocytosis, pursuant progressive expansion of innate immunity in the retina and subsequent drusen formation in Bruck’s membrane finally result in adult onset rod-cone dystrophy.

The unbiased nature of WES facilitates all protein dynamics to be scrutinized for association of gene variants with novel phenotypic demonstration. Previous reports have noted the existence of retinopathy accompanied with recurrent venous thromboses in patients with PS deficiency, mainly due to thrombosis-mediated retinal veno-occlusion, starting very early in utero. In the case of our patients, it is hypothesized that the mechanism of pathogenicity is different. In fact, degeneration of photoreceptors begins sometime after birth due to dysregulation of innate immunity as a sequel of impaired efferocytosis. Taken together, based on co-segregation of this mutation, particularly in two unrelated families, an intriguing similar RP-like phenotype and the intimate interaction of PS and MerTK in maintaining photoreceptor and RPE homeostasis, led the role of some mutations in the PROS1 gene in the pathogenesis of RP to be raised.
